# Integrating smoking cessation and alcohol use treatment in homeless populations: study protocol for a randomized controlled trial

**DOI:** 10.1186/s13063-015-0858-z

**Published:** 2015-08-29

**Authors:** Olamide Ojo-Fati, Florence John, Janet Thomas, Anne M. Joseph, Nancy C. Raymond, Ned L. Cooney, Rebekah Pratt, Charles R. Rogers, Susan A. Everson-Rose, Xianghua Luo, Kolawole S. Okuyemi

**Affiliations:** Department of Family Medicine and Community Health, University of Minnesota Medical School, 717 Delaware Street SE, Suite 166, Minneapolis, MN 55414 USA; Program in Health Disparities Research, University of Minnesota Medical School, 717 Delaware Street SE, Suite 166, Minneapolis, MN 55414 USA; Department of Medicine, University of Minnesota Medical School, 717 Delaware Street SE, Suite 166, Minneapolis, MN 55414 USA; Department of Psychiatry, University of Minnesota Medical School, Medical School, Academic Affairs, 420 Delaware Street SE, Minneapolis, MN 55455 USA; Department of Psychiatry, Yale University School of Medicine, 300 George Street, Suite 901, New Haven, CT 06511 USA; Division of Biostatistics, School of Public Health, University of Minnesota, A460 Mayo Building, MMC 303, 420 Delaware Street SE, Minneapolis, MN 55455 USA

## Abstract

**Background:**

Despite progress in reducing cigarette smoking in the general U.S. population, smoking rates, cancer morbidity and related heart disease remain strikingly high among the poor and underserved. Homeless individuals’ cigarette smoking rate remains an alarming 70 % or greater, and this population is generally untreated with smoking cessation interventions*.* Furthermore, the majority of homeless smokers also abuse alcohol and other drugs, which makes quitting more difficult and magnifies the health consequences of tobacco use.

**Methods/Design:**

Participants will be randomized to one of three groups, including (1) an integrated intensive smoking plus alcohol intervention using cognitive behavioral therapy (CBT), (2) intensive smoking intervention using CBT or (3) usual care (i.e., brief smoking cessation and brief alcohol counseling). All participants will receive 12-week treatment with a nicotine patch plus nicotine gum or lozenge. Counseling will include weekly individual sessions for 3 months, followed by monthly booster group sessions for 3 months. The primary smoking outcome is cotinine-verified 7-day smoking abstinence at follow-up week 52, and the primary alcohol outcome will be breathalyzer-verified 90-day alcohol abstinence at week 52.

**Discussion:**

This study protocol describes the design of the first community-based controlled trial (*n* = 645) designed to examine the efficacy of integrating alcohol abuse treatment with smoking cessation among homeless smokers. To further address the gap in effectiveness of evidence-based smoking cessation interventions in the homeless population, we are conducting a renewed smoking cessation clinical trial called Power to Quit among smokers experiencing homelessness.

**Trial registration:**

ClinicalTrials.gov Identifier: NCT01932996. Date of registration: 20 November 2014.

## Background

Despite progress in reducing cigarette smoking in the general U.S. population, smoking rates and related morbidity remain strikingly high among the poor and underserved. Specifically, the cigarette smoking rate remains an alarming 70 % or greater [1, 2] among the approximately 3 million homeless people in the United States the annual smoking rate among the homeless population is 70 % [3, 4]. This underserved population is not only generally unreached by smoking cessation interventions, but, in addition, tobacco-related illnesses such as lung and heart disease are fast becoming the most common cause of death in the homeless population, especially among individuals between 45 and 64 years of age [5, 6]. Given that smokers are at increased risk for tobacco-related diseases [7], such as respiratory diseases (e.g., chronic obstructive pulmonary disease and upper respiratory infection), cardiovascular diseases (e.g., stroke, hypertension and peripheral vascular diseases) and cancers (e.g., lung, head and neck, stomach, bladder and colon), continued high smoking rates in homeless populations carry serious health consequences [8–11]. Furthermore, the health consequences of smoking may be particularly increased among homeless individuals because their general health is already compromised by poor access to health care, poor nutrition and comorbidities of substance abuse, mental illness and other chronic diseases [6, 9, 12–16]. Yet, a lack of evidence on how to assist homeless smokers with quitting remains undiscovered, as smoking cessation research usually excludes this population.

In addition to high prevalence of tobacco use in homeless populations, the rates of drug and alcohol abuse are increased in this population [6]. Research has confirmed the strong association between cigarette smoking and alcohol use [17–20]. Whereas the overall cigarette smoking rates in the United States have declined, smoking rates remain significantly elevated among people with alcoholism. The prevalence of cigarette smoking among alcohol-dependent persons is nearly 80 %, more than three times the smoking rate of the general population. Cigarette smokers also drink alcohol more often and more heavily than non-smokers [21–26]. Studies have shown that the health consequences of chronic tobacco and alcohol use are synergistic [27, 28]. For instance, in one study of 4928 people with alcoholism [29], high cancer mortality was attributed solely to smoking. One potential enhancement to smoking interventions for homeless smokers is to provide concurrent alcohol treatment for those with heavy alcohol use. In studies of concurrent smoking cessation and substance use treatment, researchers have consistently found that smoking abstinence is associated with reductions in substance use after treatment [30, 31]. Despite ample evidence that pharmacotherapy and counseling are effective for smoking cessation in the general population, to date no one has identified effective methods for extending the benefits of these treatments or related interventions to the homeless.

Despite high cigarette-smoking rates and disease burden in homeless populations, smoking cessation programs have been focused, for the most part, on non-homeless persons. The exclusion of homeless smokers from smoking cessation interventions may rest on the beliefs that smokers in this population are difficult to recruit and retain in clinical trials and that the population lacks interest in cessation. Also, owing to the fact that homeless individuals are faced with meeting basic survival needs such as finding food and shelter, some may assume that smoking cessation is not a priority for the homeless and that it should not be a priority for health care providers. However, recent data do not support this assumption. In a survey of 236 homeless adults at 9 homeless service sites, researchers found a smoking prevalence of 69 %, and of these smokers, 72 % attempted to quit at least once and 37 % reported readiness to quit smoking within the next 6 months [5]. In the same study, the investigators found that nicotine replacement therapy (NRT) alone or in combination with other treatments was the most preferred treatment. Our research team [20] found that, compared with their non-homeless counterparts, homeless smokers had made similar numbers of quit attempts in the previous year and expressed similar levels of interest in participating in a program to help them quit smoking (7.4 for homeless vs. 7.9 for non-homeless on a scale of 1–10) [32].

In our recently completed Power to Quit (PTQ) study (*N* = 430) [32, 33] targeting homeless smokers, we found cotinine-verified 7-day quit rates to be 9.3 % for motivational interviewing (MI) vs. 5.6 % for the control group at 26 weeks. These quit rates are low when compared with those in the general population, demonstrating the challenge for a smoking intervention in homeless populations. A variety of factors may have contributed to low quit rates in the study, including (1) high level of nicotine dependence as indicated by mean 20 cigarettes per day and 87 % of the participants smoking their first cigarette within 30 minutes of awakening, (2) high rates of substance abuse and psychiatric comorbidities [18, 19], (3) competing daily social needs and (4) need for more cognitive behavioral strategies instead of MI, given the high baseline motivation to quit (9+ on a 1–10 scale). The low quit rates revealed in studies of alcohol-dependent and homeless smokers, along with positive outcomes in studies of combined NRT, suggest that a more intensive intervention ought to be considered. The primary aim of the present study is therefore to evaluate the effects of an intensive smoking intervention that integrates alcohol abuse treatment and smoking cessation in a homeless population. We will use a three-group randomized design to test the study hypotheses in a community-based setting. The three study conditions are (1) integrated intensive smoking intervention using cognitive behavioral therapy (CBT) plus alcohol intervention (IntS+A), (2) an intensive smoking (IS) intervention using CBT or (3) usual care (UC; brief smoking cessation and brief alcohol counseling, both based on U.S. Public Health Service [USPHS] guidelines) [34, 35]. In addition, all participants will receive a 12-week treatment with a combination of nicotine patch plus gum or lozenge.

## Methods/Design

### Overview of study design

**Fig. 1:**
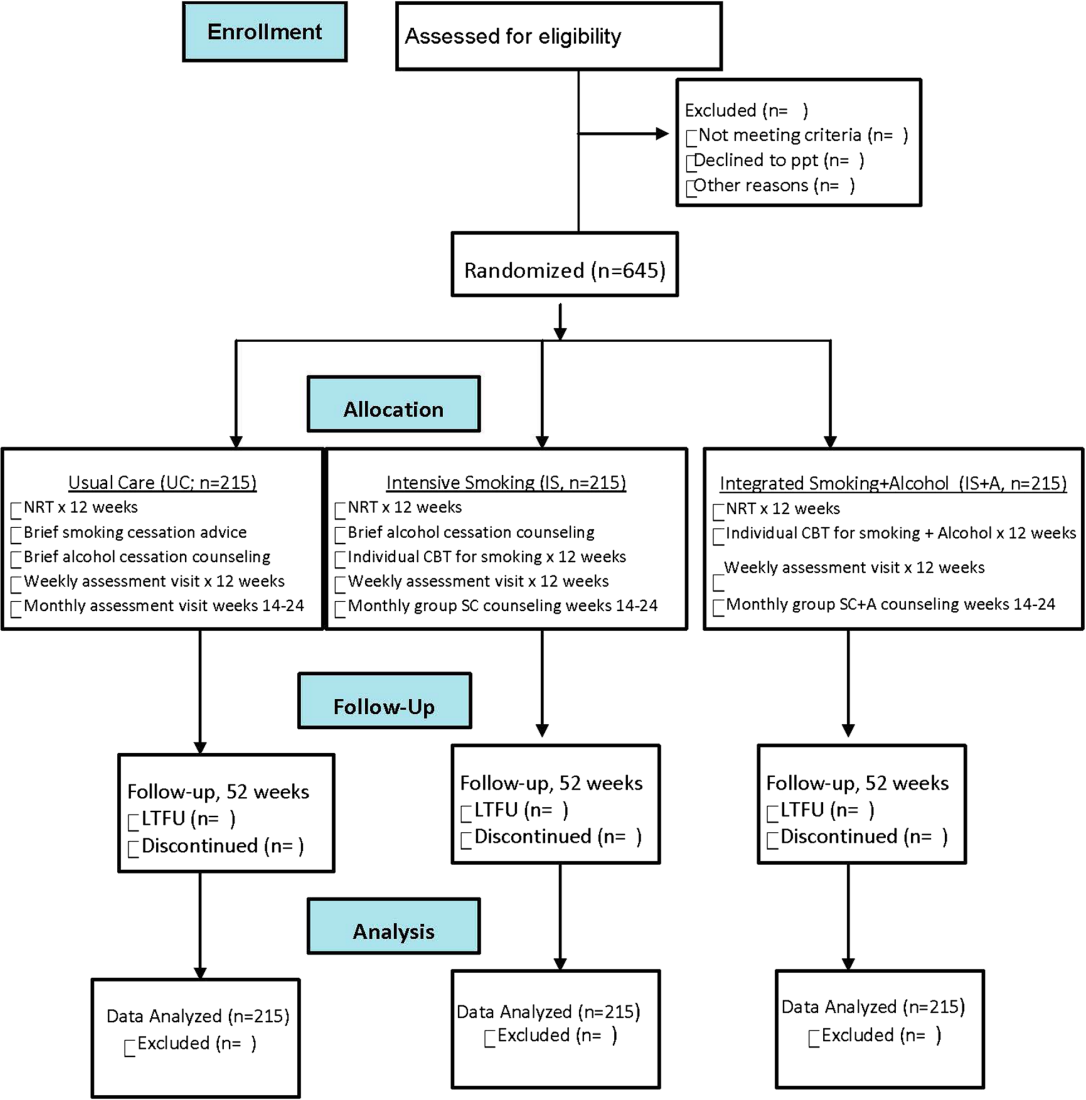
Flowchart of the Enhancing Smoking Cessation in the Homeless Population study. *CBT* cognitive behavioral therapy, *NRT* nicotine replacement therapy, *LTFU* long-term follow-up, *SC+A* combined smoking cessation and alcohol

Figure 1 gives an overview of the study. The primary aim of the current PTQ-2 study is to evaluate the effects of an intensive smoking intervention that integrates alcohol abuse treatment in a homeless population. Eligibility screening and all subsequent study visits will take place at homeless shelters to make participation convenient for participants. Once eligibility is determined, participants will complete a baseline assessment. Informed consent will be obtained from each participant before any study procedures are initiated. Participants will be randomized to one of three groups: UC (NRT + brief, one-time counseling for both alcohol and smoking cessation), IS (NRT+CBT for smoking cessation) plus brief alcohol counseling, and IntS+A (NRT+CBT for both alcohol and smoking cessation). All three groups will receive biweekly supplies of NRT (21-mg patch + gum and/or lozenge) for a total of 12 weeks beginning at week 4. Participants in all three groups are instructed on the correct daily use of both the patch and gum/lozenge and are encouraged to contact study staff with any concerns or issues related to NRT use. The UC group will receive a brief, one-time counseling session (approximately 20 minutes) of standard advice for both alcohol and smoking cessation at their baseline visit, which is based on the USPHS guidelines [34].

Counseling using CBT follows a protocol used in a recent study of alcohol-dependent smokers [36–38]. During the first 3 weeks after enrollment, CBT counseling sessions will focus on the participants’ preparation for their quit date, on which they will receive the NRT. Participants randomized to the IS will receive brief alcohol counseling plus 12 weekly CBT counseling sessions, starting at their baseline visit, that are 20–30 minutes in length. Last, participants randomized to the IntS+A group will receive 12 weekly CBT counseling sessions beginning at their baseline visit, each of which are approximately 50–60 minutes in length. The study design does not include a placebo arm, owing to ethical concerns. The primary smoking outcome is cotinine-verified 7-day smoking abstinence at follow-up week 52, and the primary alcohol outcome will be breathalyzer-verified 90-day alcohol abstinence at follow-up week 52. The secondary smoking outcome will be prolonged smoking abstinence at weeks 12, 26 and 52 with CO verification at weeks 12 and 26. CO is used for verification at these times because cotinine verification is not useful in the context of NRT. The secondary alcohol outcome will be self-reported continuous alcohol abstinence for 30 days at weeks 12 and 26 and for 90 days at week 52.

### Study setting

All study procedures, including eligibility, enrollment, assessments and counseling sessions, will be administered at the homeless shelters. Participants will be recruited from a variety of homeless emergency shelters and transitional housing units in the Minneapolis-St. Paul (Twin Cities), MN, USA, area. Currently, participants for the PTQ-2 pilot are being recruited from the Dorothy Day Center in St. Paul, MN, USA, with expansion to other sites planned for the initiation of the main study (see Table 1). Strategies for recruiting participants begin with establishing connections with potential shelter sites and conducting informational sessions with shelter staff. Recruitment then progresses to include promotional flyer distribution, announcement of the PTQ-2 study at peak use times at the shelter and word of mouth from current participants and study staff to shelter users. The study team will include the project coordinator, three full-time research counselors with a minimum of a master’s degree plus at least 1 year of experience in CBT for addiction treatment, two volunteers who will assist the community mobilizer (outreach workers) in recruiting and enrolling participants, and a community mobilizer who has a past history of homelessness and working with the homeless population. The volunteers will also assist the team in administering the eligibility surveys to the willing participants. All research counselors will receive extensive CBT training from the licensed clinical psychologist on the research team before the commencement of the project. The project office will serve as the base location for the research team and will be located at a facility nearby to the homeless shelter. The project coordinator will review the surveys before enrolling the eligible participants.

**Table 1:** Community-based shelters and transitional housing facilities, population served, services offered and average length of stay

Community-based research site	Overview of guests’ demographics	Services offered	Average length of stay (days)	Persons served/yr
Dorothy Day Center (DDC)	The DDC is run by the umbrella organization Catholic Charities of Minnesota. The population that uses DDC is mostly male and mostly single; 15 % of the guests are over 55 years of age.	Emergency shelter, space for 250	90	2688/2014
		Transitional housing, 42-bed women’s shelter		
		Meals (breakfast, lunch, dinner)		
		Food shelf		
		Medical and mental health care		
		Housing and employment advocacy		
		Counseling support		
		Laundry and bathroom/shower facilities		
		Computer laboratory		
Our Savior’s Shelter (OSS)	OSS offers faith-based activities and other services. In the emergency shelter program, 85 % of the individuals are male, 46 % are African American and 88 % have a high school diploma or graduate equivalency degree (GED) or more. In the transitional housing program, there are 15 men, 16 women and 9 children.	Emergency shelter, 40 beds per night	34	650/2013
		Transitional housing, 40 men, women and children		
		Two meals daily (lunch, dinner)		
		Bathroom/shower facilities		
		Case management services		
		Permanent supportive housing services		
Union Gospel Mission	The Union Gospel Mission site is a faith-based site that concurrently runs a short-term emergency relief program alongside a transitional housing program and an intense alcohol and drug treatment facility.	Emergency shelter, space for 120 persons	32	2015
		Transitional housing, 145 rooms		
		Alcohol drug treatment center, 75 men		
		24-h service desk		
		Intensive treatment programs		
		Bible study and work therapy		
		Large-group service		
		Health care/dental services		
		Clothing giveaway		
		Spanish interpreters		
People Serving People (PSP)	PSP is a short-term transitional housing center that is geared to helping families find housing. The program’s stated goal is to help children by giving them direct care or by assisting their parents to gain the skills that will keep their families off the streets. The program serves women and children. About 60 % of those PSP serves are under 18 years of age, and 32 % of the total population is under age 5 years.	Three meals daily	39	1,286/2013
		Medical clinic open 5 days/week		
		Mental health and chemical dependency services, employment assistance, literacy, early childhood development and legal aid programs		
		Guest access to voice mail, e-mail and in-house library		
		24-h front desk service supplying diapers, formula, warm clothing and other basic needs		
		Training programs in life skills, culinary arts, facilities training and workforce development		
		Counseling services		
		Family advocates to ensure families get connected with the resources and services they need		

During enrollment, participants will be given the Health Insurance Portability and Accountability Act of 1996 (commonly known as “HIPAA”) and consent forms to read carefully and sign if they wish to be enrolled in the trial. The staff will ensure that the participants have full understanding of the content of the forms by asking open-ended questions before they sign the documents. Baseline surveys, CO and breathalyzer tests, sputum collection and randomization will be administered by the community mobilizers in the cafeteria within the shelter, away from the shelter officers and other participants to maintain confidentiality. On the basis of the experience in the first PTQ study, we believe that this entire process should last approximately 90 minutes. The counselors will then take the enrolled participants to a private office reserved by the shelter staff for a one-to-one counseling session to establish a counselor–participant relationship and reassure participants of the privacy and confidentiality of their participation. Each counseling session will take between 30 and 60 minutes, depending on to which randomized arm the participant is allocated. To stay organized, staff will create a separate file for each enrolled participant. Each file will consist mainly of the counselors’ materials and other records pertaining to the participants. Reminder calls will be made and appointment reminder slips will be given to participants. An adverse event worksheet will also be completed by the staff when a participant cannot make the appointment because of an illness or injury. This system was effective in the last PTQ study and contributed to the 75 % follow-up and retention rate.

### Participants

**Fig. 2:**
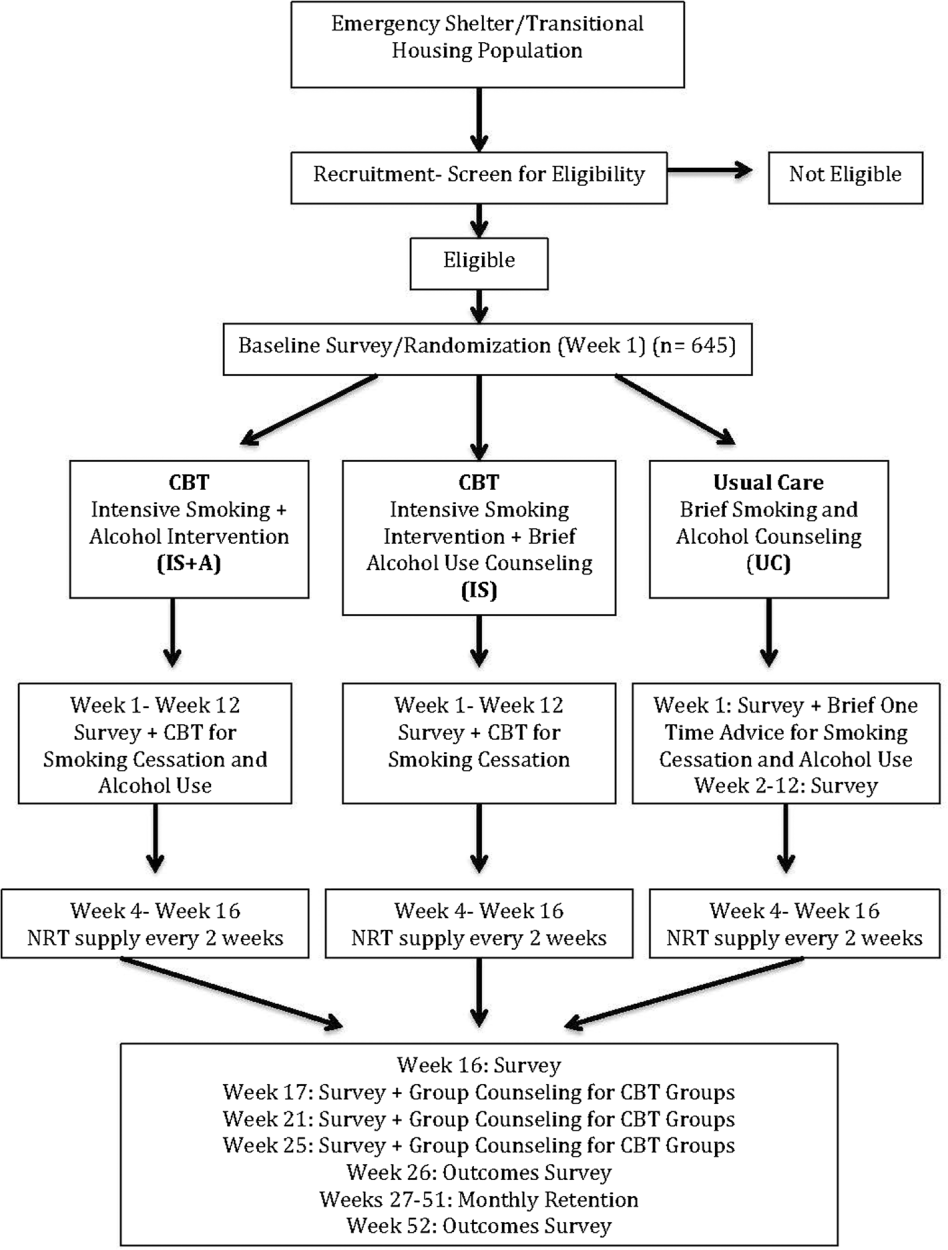
Overview of study procedures. *CBT* cognitive behavioral therapy, *NRT* nicotine replacement therapy

A total of 645 participants will be enrolled in the study (Fig. 2). Final follow-up for each participant will be at week 52 of their participation in the trial. Participants will be randomized into one of three groups: UC (*n* = 215), IS (*n* = 215) or IntS+A (*n* = 215). Eligibility criteria are (Table 2) currently homeless [21], current daily cigarette smoker, aged 18 years or older, willing to use NRT (nicotine patch and gum or lozenge) for 12 weeks, willing to participate in counseling sessions for 12 weeks and willing to complete 16 total appointments (12 weekly counseling visits, 3 monthly group counseling visits and 1 retention visit) over the 52-week study period. The determination of participants’ homelessness will be based on the Stewart B. McKinney Act, passed by the U.S. Congress in 1987, in which *homelessness* is defined as “any individual who lacks a fixed, regular and adequate nighttime residence” or “one whose primary nighttime residence is a supervised publicly or privately operated shelter designed to provide temporary living accommodations, transitional housing, or other supportive housing program or a public or private place not meant for human habitation (e.g., on the streets or in abandoned buildings, tents, or automobiles)” [4, 9, 21]. Participants will also be considered to be homeless if they have been residing with family or friends for a period of 3 months or less. *Current smoking* is defined as smoking at least one cigarette per day in the previous 7 days and having smoked at least 100 cigarettes in the participant’s lifetime. To confirm their smoking status, participants will be asked to take an exhaled CO test with a reading of at least 5 parts per million, based on recommendations of the Society for Research on Nicotine and Tobacco [22–24].

**Table 2:** Eligibility criteria for participants

Inclusion criteria	Exclusion criteria
• Currently homeless^a^	• Participation in previous PTQ study
• Smoke ≥1 cigarette/day in previous 7 days	• Use of smoking cessation medications or interventions in previous 30 days
• Smoked ≥100 cigarettes in lifetime	• Unstable medical illness that requires immediate medical care
• AUDIT score ≥7	• AUDIT score <7
• Aged 18 years or older	• Pregnancy or other NRT contraindications
• Willing to attend study sessions and follow other study protocols	• Current history, or in past 6 months, of psychotic disorder or major depressive disorders and not stable on treatment for past 3 months^b^
	• Cognitive impairment^c^

*AUDIT* Alcohol Use Disorders Identification Test, *NRT* nicotine replacement therapy, *PTQ* Power to Quit

^a^We will use the definition in the Stewart B. McKinney Act, passed by the U.S. Congress in 1987

^b^This determination will be made by the study psychiatrist

^c^We will use the Mini Mental State Examination to make this determination

Additional eligibility criteria include willing to use NRT, having lived in the Twin Cities area for at least 3 months and planning to stay in the Twin Cities area for the next 12 months. Additionally, women are excluded if they are currently pregnant or are not willing to use birth control for the duration of the study. Exclusion criteria include use of smoking cessation medications or interventions in the previous 30 days, unstable medical illness that requires immediate medical care, current history of alcohol dependence, previous severe alcohol withdrawal symptoms and/or events, Alcohol Use Disorders Identification Test score less than 7, pregnancy and cognitive impairment.

A community advisory board (CAB) with 10 members will be convened before the launch of the PTQ-2 pilot. The plan is for the CAB to meet two or three times each year during the 5-year project. The CAB, which is an important part of the study, is composed of directors and managers from various shelters and agencies throughout the Twin Cities area that serve homeless individuals. The CAB plays a crucial role in providing insight into the challenges homeless individuals faced, as well as barriers related to study participation and retention. CAB members also contribute valuable insight that is helpful in aiding study activities with this vulnerable population.

### Randomization process

After final eligibility is confirmed, all participants who sign the informed consent document will be randomized into one of three groups: (1) IntS+A, (2) IS or (3) UC. A block randomization with three or six random blocks will be used to improve balance. The statistician developed the randomization scheme for the study. In this scheme, each participant is randomized on-site by study staff using the established protocol during the enrollment visit. All participants will receive 12-week treatment with nicotine patch plus nicotine gum and/or lozenge.

### Counselor training

Our therapists do not come to the trial as trained psychotherapists, but they are provided with extensive CBT training before engaging with participants. Although our counselors are trained in CBT, they are not trained psychotherapists. Counselors meet individually or in groups of two for intensive training with the counseling supervisor for a minimum of 40 hours before being assigned participants. In addition, fidelity monitoring and supervision are provided each week in a group format with individual supervision provided on an as-needed basis.

### Intervention components

#### Integrated intensive smoking intervention using cognitive behavioral therapy plus alcohol intervention

Participants in the IntS+A group will receive separate alcohol and smoking counseling during each session. Each session will be broken down into two 30-minute segments of distinct alcohol counseling and smoking intervention. Participants will receive weekly counseling for the first 3 months, followed by monthly group sessions for the next 3 months. After the initial session, the counselor will allow the participant to decide whether to address alcohol or smoking first, and each subsequent session will alternate which topic begins the session. The content covered in the smoking module in the IntS+A condition is identical to that in the IS condition. The CBT model employed to address both smoking and alcohol follows the antecedent–behavior–consequences) model and places equal weight on identifying and changing thought patterns as behavior modification. Counselors will encourage participants to identify their antecedent thoughts and situational factors (i.e., triggers and cues) contributing to their smoking and alcohol use and to examine the consequences of their use. The alcohol counseling will be distinct from smoking counseling, and in it similarities between quitting smoking and stopping alcohol and drug use will not be emphasized. This is based on findings from a study [39] in which researchers reported that emphasizing these similarities led to worse alcohol outcomes compared with concurrent treatment that did not emphasize the similarities. The alcohol intervention manual is based on the National Institute on Drug Abuse manual for CBT for drug abuse [38, 39]. The treatment manuals and participant handbooks were developed using both the alcohol treatment protocol used by Cooney and colleagues [38] and a CBT approach [38, 40]. Both manuals were modified to address the needs of this trial. The key alcohol interventions will include self-monitoring of triggers, cravings and urges; and functional analysis of drinking behavior (i.e., antecedents, behavior and consequences).

#### Intensive smoking intervention using CBT

Participants in the intensive smoking intervention arm will receive 12 weeks of NRT plus weekly individual smoking cessation counseling sessions for the first 3 months, followed by monthly group sessions for the next 3 months. In addition, they will receive the brief alcohol counseling also provided to the UC participants. CBT strategies will be employed to identify and address smoking triggers and to employ urge reduction strategies, including delay, escape, avoid, distract and substitute. The key intervention strategies will also include introducing self-monitoring of smoking behavior, completing a functional analysis of smoking using a smoking “wrap sheet,” developing a specific quit plan, enlisting allies and relapse prevention. The participants will be asked to quit after their fourth counseling session [41].

#### Usual care (brief smoking cessation and brief alcohol counseling, both based on USPHS guidelines)

In the UC condition, participants will receive NRT as described above for the IS intervention and brief (20 min), one-time counseling for smoking and alcohol cessation. The smoking cessation counseling will be based on the “five A’s” model (ask, advise, assess, assist, arrange) recommended in the USPHS clinical practice guidelines [34]. Consistent with these guidelines, counselors will carry out the following procedures: (1) congratulate the participant on enrolling in the study, (2) describe the harms related to smoking and the benefits of quitting, (3) advise the participant to quit immediately, (4) assist participants to set a quit date, (5) encourage participants to tell friends and family about their quitting and (6) describe potential relapse situations and other barriers to quitting. The brief alcohol counseling will follow USPHS guidelines [42] and includes elements such as (1) presenting screening results, (2) identifying risks, (3) discussing consequences, (4) soliciting commitment, (5) identifying goals and (6) giving advice and encouragement. At the end of the session, participants interested in additional smoking or alcohol interventions will be referred to local and national treatment programs. We will measure participants’ engagement in smoking cessation or alcohol programs outside the study as potential moderators of intervention effects.

### Study procedures

#### Individual session 1

The first session will begin at baseline after enrollment. This visit will last up to 90 minutes, whereas most other visits will last 30–60 minutes, depending on to which group the participant is assigned. Ground rules will be established at the first counseling session; participants will also be informed that each counseling session will be audio-recorded before the commencement of the session. CO testing and an alcohol breathalyzer test will be conducted at all individual sessions. The UC group will receive a brief, one-time advice session on smoking cessation and alcohol use. Participants will receive a $20 gift card, tote bag and two bus tokens at this session as compensation for their time.

#### Individual sessions 2–12

Session counseling procedures for the IS and IntS+A conditions will follow the same format as the first session. At all sessions, a survey will be administered by the community mobilizer before the counseling session begins. Additionally, participants will be asked about their smoking history and/or drinking patterns, as well as craving, depending on the CBT arm to which they were randomized. Task assignments, within-session role playing, coping skills training, identification of cognitive and environmental antecedents, behavioral choices and consequences, and both past and future high-risk situations will be carried out during the CBT counseling sessions. A quit date will be set in the smoking arm at week 4. NRT (nicotine patch plus gum or lozenge) will also be carried out for 12 weeks starting at week 4. The estimated session durations are 30 minutes for the IS group and 60 minutes for the IntS+A arm.

#### Group sessions 17–25

After the week 16 survey, participants in the CBT groups will be asked to return for booster group sessions for 3 months. Reminder calls will be made during the week to each participant. Participants will be asked to complete the weekly survey at their arrival. They will be encouraged to discuss their abstinence status and about the day-to-day challenges they encounter. The research counselors will ensure that the environment is comfortable for the participants and conducive to their expressing themselves. Procedures for the group will follow the CBT format covered during the individual sessions. All group counseling sessions will be cofacilitated by trained study and homeless shelter staff. Participants will receive a $10 gift card and two bus tokens for as compensation for their time. The estimated duration of the sessions is 30–60 minutes.

#### Weeks 26 and 52 follow-up

Participants will be asked to return at 6 months and 12 months postbaseline to participate in an outcome survey and cotinine analysis to verify abstinence status. After collection of samples and assessment, participants will be asked about the study program and how to improve it. Participants will receive $50 for participation at week 26 and $75 for week 52. Each visit will take approximately 60–90 minutes.

### Outcomes

The primary smoking outcome is cotinine-verified 7-day smoking abstinence at follow-up week 52, and the primary alcohol outcome will be breathalyzer-verified 90-day alcohol abstinence at week 52. Exploratory aims are to evaluate (1) how smoking cessation or reduction relates to psychosocial factors such as levels of depression, hopelessness and perceived stress at weeks 12, 26 and 52 of follow-up; (2) how alcohol abstinence or reduction relates to psychosocial factors such as levels of depression, hopelessness and perceived stress at 12, 26 and 52 weeks of follow-up; (3) how treatment outcomes relate to history of other substance abuse; (4) how treatment outcomes relate to opportunities for employment and housing, as well as overall health and well-being; and (5) participants’ perceptions of the acceptability and usefulness of components of the various interventions. Additional aims include identifying potential impacts on intervention efficacy of psychosocial factors such as depression, hopelessness and perceived stress; other substance abuse; changes in employment and housing; and overall health.

### Participant incentives

Participants will be compensated with non-monetary incentives such as tote bags, bus tokens, stress balls, pens and water bottles after taking the eligibility survey. At enrollment, each participant will receive a $20 debit card serviceable at any automated teller machine within the United States, in addition to other small gift items, such as playing cards, $5 Subway gift cards, bus tokens and tote bags. Participants will receive $50 for participation at week 26 and $75 for week 52. For all other visits completed, participants will receive monetary incentives of $10. Participating shelters will also be compensated up to $1000/year for their assistance with recruitment and retention. Shelters will receive separate payments for their therapists who participate in training ($25/h) and cofacilitate the group counseling sessions ($50/h).

### Data management

Data management for this project will encompass data entry, data cleaning, identifying and tagging any crossovers and conversion into proper format for data analysis and recoding. REDCap (Research Electronic Data Capture; http://project-redcap.org/), a secure web application for building and managing online surveys and databases, will be used for design, implementation and maintenance of the database. In addition, a computer-based tracking system will be developed to follow each patient and to prompt the staff regarding the upcoming data collection point. Data collection points for each subject will be calculated from his or her initial date of contact. Before data collection, the study protocol was approved and monitored by the University of Minnesota’s Institutional Review Board.

### Statistical analyses and sample size and power calculations

For this study, 645 participants will be randomized at baseline to test the main effects of smoking and alcohol interventions for the primary smoking outcome (cotinine-verified 7-day smoking abstinence at week 52 follow-up) and alcohol outcome (breathalyzer-verified continuous alcohol abstinence for 90 days at week 52 follow-up). The study is powered to test the primary hypotheses using χ^2^ tests and the secondary hypothesis using Fisher’s exact test. Assuming a two-sided χ^2^ test with a type I error rate of 0.025 for each of two primary outcomes, smoking abstinence rates of 18 % in the IntS+A group versus 8 % in the IS group and 4 % in the UC group, a sample size of 215 participants in the IntS+A group and 430 participants in the IS and UC combined group will achieve a power of 80 %. With 645 participants and a type I error rate of 0.025, we have greater than 90 % power for the primary alcohol hypothesis, assuming 90-day alcohol abstinence rates of 32 % vs. 10 % for the IntS+A group versus the IS intervention or UC groups . Assuming a two-sided Fisher’s exact test with a type I error rate of 0.05 for the secondary outcome of smoking abstinence rate of 12 % in the IntS group versus 4 % in the UC group, a sample size of 215 participants per group will achieve a power of 82 %. Our sample size calculation was based on the analysis that classified those lost to follow-up as smokers or drinkers and therefore has accounted for up to a 25 % attrition rate observed in the prior Power to Quit (PTQ) study [32, 33].

#### Basis for sample size

We based our estimates for the main outcomes on data from our previous PTQ study among homeless smokers, as well as from the available literature [38, 43]. In the PTQ study, 7-day abstinence rates at week 26 were 9.3 % for the MI+NRT condition and 5.1 % for the control group. In another study [43] of alcohol-dependent smokers, our research team reported 7-day abstinence rates at 6 months as 9.1 % and 2.2 % for an intensive smoking intervention (three counseling sessions + NRT) and brief advice, respectively. We therefore projected the smoking abstinence rates as 4 % for the UC condition and 12 % for the IS condition. Our PTQ study also showed that the smoking abstinence rate for those who quit drinking during the study was nearly double that for drinkers, despite not having an alcohol intervention. We conservatively projected the smoking abstinence rate for the IntS+A condition as 18 %. We based the projection for the alcohol abstinence rates on a study [38] in which our research team found 90-day alcohol abstinence rates of 32–43 % at 12 months among alcohol-dependent smokers.

### Data analysis

Before initiating outcome analyses of quantitative data, we will examine distributions for all variables, with particular attention to variable ranges, missing values, skewness and transforming variables when indicated according to the criteria of Winer [44]. Using analysis of variance (ANOVA) for continuous data and χ^2^ tests for categorical data, we will examine group comparability at baseline to determine whether randomization was successful in creating equivalent groups with regard to demographic, process and important independent variables such as education, income and literacy levels. These data will help determine if there is a need to incorporate stratifying variables or covariates into later analyses.

#### Analyses for primary hypotheses

The primary analysis will be based on an intention-to-treat analysis; that is, participants will be analyzed regarding the treatment to which they were randomized. The intervention effects for the primary outcomes of week 52 biochemically verified 7-day abstinence from cigarette smoking and week 52 ninety-day abstinence from alcohol consumption will be tested using χ^2^ tests. Any participants lost to follow-up will be classified as treatment failures (i.e., smoker or drinker). Bonferroni procedures will be used to control for experiment-wise type I error rate. Abstinence rates and corresponding 95 % confidence intervals will be estimated and summarized using appropriate tabular and graphical methods.

In supportive analyses, we will examine baseline variables, including demographics (age, sex, income and education), nicotine dependence, withdrawal symptoms, motivation and confidence, self-efficacy, social support, perceived stress and depressive symptoms, in terms of their relationship to study outcomes. Analyses accounting for these measures will be conducted using multiple logistic regression including intervention groups as controlled factors. Thus, we will be able to assess whether the main conclusions from the primary analysis are robust for the inclusion of these baseline variables. Fisher’s exact test will be used to compare the biochemically verified 7-day smoking abstinence rates at week 52 follow-up between the IS and UC participants. Supportive analysis will be conducted using multiple logistic regression including the intervention group as a predictor, adjusting baseline covariates to assess whether the result from secondary analysis is robust for the inclusion of baseline covariates.

#### Analyses for exploratory aims

To examine how smoking cessation, alcohol abstinence, smoking reduction and alcohol reduction at follow-up weeks 12, 26 and 52 relate to the psychosocial factors (e.g., level of depression, hopelessness and perceived stress), other substance abuse, opportunities for employment and housing and overall health, separate repeated-measures logistic regression analyses with generalized estimating equations or linear mixed models that account for the repeated-measures structure of the data will be used to examine the associations between each individual psychosocial predictor and smoking or alcohol outcomes at follow-up weeks 12, 26 and 52. Interaction terms between these factors and intervention indicators will be further tested in these models. In model validation, we will use analytic and graphical techniques to check assumptions of linearity, homoscedasticity, multivariate normality and independence of residuals. Descriptive statistics will be used to summarize data, and ANOVA or χ^2^ tests will be used to examine participants’ perceptions of the acceptability and usefulness of components of the various interventions.

### Ethics

Ethical approval was received from the University of Minnesota’s Human Research Protection Program (approval date: 30 July 2014; Institutional Review Board code number: 1307 M39761; assurance of compliance number FWA00000312 [Fairview Health Systems Research FWA00000325, Gillette Children’s Specialty Healthcare FWA00004003]). The trial is registered at ClinicalTrials.gov (NCT01932996).

## Discussion

This study protocol describes the design of the first community-based controlled trial (*N* = 645) to examine the efficacy of integrating alcohol abuse treatment with smoking cessation as an intervention for homeless smokers. The study has a three-arm randomized design to test the effects of intensive smoking intervention (i.e., higher dose and duration than the previous PTQ), and alcohol abuse treatment is integrated with smoking cessation using CBT. The three study conditions are an integrated intensive smoking intervention using CBT in addition to an alcohol intervention (IntS+A); an intensive smoking intervention using CBT (IS); and usual care comprising brief smoking cessation and brief alcohol counseling, both based on USPHS guidelines (UC). In addition, all participants will receive 12-week treatment with a combination of nicotine patch plus gum or lozenge. As a strategy to integrate the proposed project with existing programs at the shelters, all group counseling sessions will be cofacilitated by trained study homeless shelter staff. The previous study (*N* = 430) targeting homeless smokers, although adequately powered, was a relatively low-intensity study that showed cotinine-verified 7-day quit rates of 9.3 % for MI and 5.6 % for brief advice at 26 weeks. The investigators also found that quitting smoking was associated with reduced alcohol use. These smoking quit rates are low compared with the general population, demonstrating the challenge in delivering tobacco interventions in this population and the need for more research. Further, the two study conditions (8.7 % vs. 17.4 %) did not reach significance. The lack of a significant difference in treatment effect size at 26 weeks was attributed to the short duration of the intervention. Therefore, in the present study, we will examine the feasibility of enrolling and retaining a group of homeless smokers in a 12-week smoking cessation program that combines individual counseling based on CBT principles with pharmacotherapy (nicotine patch and gum or lozenge) at week 52. The rationale for using the CBT intervention rather than the MI intervention is that baseline data showed that participants were already highly motivated to quit smoking, as evidenced by the 75 % retention rate at the week 26 final visit [32], and that they could therefore benefit from CBT strategies. The designing of this study includes more intense interventions that include a higher dose of pharmacotherapy (nicotine patch combined with nicotine gum or lozenge for 12 weeks), an increased number of counseling sessions weekly for 3 months, an increased duration of individual sessions from 15–30 minutes to 60 minutes and use of CBT to provide more cognitively based strategies in the counseling sessions. Many of the challenges researchers faced in the previous clinical trial, such as those seen when recruiting, retaining and intervening with a homeless population, were adjusted for in the design of this study. Challenges included high migration within the region as well as between shelters within the Twin Cities area, competing needs that made it difficult to keep appointments, and limited forms of available communication. The study was designed with minimal exclusion criteria so that the external validity of the results would be enhanced. Understanding the effectiveness of smoking cessation and alcohol abuse treatment for this underserved population will assist researchers and health care providers in developing and implementing smoking cessation interventions for homeless and other vulnerable populations. The primary smoking outcome is cotinine-verified 7-day smoking abstinence at follow-up week 52, and the primary alcohol outcome is breathalyzer-verified 90-day alcohol abstinence at week 52.

## Trial status

The study is still in the first phase of participant recruitment and enrollment.

## Abbreviations

AUDIT: Alcohol Use Disorders Identification Test
CAB: Community advisory board
CBT: Cognitive behavioral therapy
CO: Carbon monoxide
DDC: Dorothy Day Center
HIPAA: Health Insurance Portability and Accountability Act of 1996
IS: Intensive smoking
IntS+A: Integrated smoking and alcohol
LTFU: Long-term follow-up
MI: Motivational interviewing
NRT: Nicotine replacement therapy
OSS: Our Savior’s Shelter
PSP: People Serving People
PTQ: Power to Quit
SC+A: Combined smoking cessation and alcohol counseling
UC: Usual care
USPHS: U.S. Public Health Service
